# Psychological Sciences section of The Journals of Gerontology Series B: Featured 2024 Editor’s Choice Articles

**DOI:** 10.1093/geroni/igaf122.1304

**Published:** 2025-12-31

**Authors:** Duke Han

## Abstract

The psychological sciences of the aging process are vast in scope and complexity. Methodological approaches that are interdisciplinary, innovative, and inclusive can make these enigmatic processes more tangible. This symposium showcases articles in the Psychological Sciences section of The Journals of Gerontology, Series B: Psychological Sciences and Social Sciences that were selected as Editor’s Choice in 2024 because they exemplify these ideals. Bey et al. presents evidence on the interaction between psychosocial risk and neighborhood disadvantage among older Black adults. Huo et al. provides evidence for associations between caregiver ambivalence and psychological and cognitive symptoms of patients with Alzheimer’s. Doyle et al. sheds light on characteristics of Hispanic SuperAgers. Ly et al. presents evidence for the impact of discrimination on metamemory.

